# Genetic Diversity of *Mycobacterium tuberculosis* Isolates from Inner Mongolia, China

**DOI:** 10.1371/journal.pone.0057660

**Published:** 2013-05-01

**Authors:** Qin Yu, Yunkai Su, Bing Lu, Yan Ma, Xiuqin Zhao, Xiaomin Yang, Haiyan Dong, Yao Liu, Lulu Lian, Li Wan, Yimou Wu, Kanglin Wan

**Affiliations:** 1 National Institute for Communicable Disease Control and Prevention, Chinese Center for Disease Control and Prevention/State Key Laboratory for Infectious Disease Prevention and Control, Beijing, P. R. China; 2 Pathogenic Biology Institute, University of South China, Hengyang, Hunan Province, China; 3 Inner Mongolia institute for Tuberculosis Control and Prevention, Hohhot, China; Institut de Pharmacologie et de Biologie Structurale, France

## Abstract

**Background:**

Tuberculosis (TB) is a serious public health problem in China, and within China, Inner Mongolia has a high prevalence area of TB. Though studies on the genetic diversity of *Mycobacterium tuberculosis* (MTB) have been reported in many provinces, there are no such studies to date in Inner Mongolia. In this study, we investigated the genetic diversity of MTB in Inner Mongolia.

**Methodology/Principal Findings:**

In this study, we analyzed 372 clinical MTB isolates with 22-loci mycobacterial interspersed repetitive unit and variable-number tandem repeats (MIRU-VNTR), spoligotyping, large sequence polymorphism (LSP), and NTF region analysis to understand the TB genotypes prevalent in Inner Mongolia. We found that the Beijing family was the most prevalent genotype (85.48%, 318/372), and the “modern” sublineage accounted for 76.73% (244/318) of the isolates. Our data also showed that there was no statistically significant association between the two major nationalities and the Beijing genotype (χ^2^ = 3.612, P = 0.057; P>0.05).

**Conclusion/Significance:**

The Beijing genotype is the most prevalent family of *M. tuberculosis* in Inner Mongolia, and we do not find any correlation between the Beijing genotype and the major nationalities.

## Introduction

Tuberculosis (TB) is a major public health problem that has threatened the health of human beings worldwide, especially in developing countries. Although the World Health Organization (WHO) has launched the “Global Plan to Stop Tuberculosis”, which aims to save a million lives by 2015 [1], China, the second among the 22 high-burden countries, has different incidences and prevalence of TB in different provinces.

Inner Mongolia is located in the northern China border area, adjacent to southern of Mongolia and Russia. There are 23.79 million people of 49 minorities living in this region of 1.10 million square kilometers, making it the third largest populated area in China. Based on the 1990 National TB Epidemiology Survey in China, the prevalence rate of TB in Inner Mongolia was the third, following Tibet and Sichuan, and higher than that in this region in 1979 [2]. Moreover, there is as yet little information about the molecular epidemiology of tuberculosis in Inner Mongolia, so there is an urgent need for studies addressing the molecular epidemiology of TB and/or TB genotyping in this area.

To investigate the spreading features and track transmission chain of TB, to detect suspected outbreaks, and to determine the genetic relationships among the *Mycobacterium tuberculosis* (MTB) strains isolated from TB patients in this area, a suitable molecular typing method of MTB strains has been proven to be important for TB control [3]. Based on the DNA polymorphisms of MTB and advances in PCR techniques during the last few years, some genotyping methods have been widely used in MTB molecular typing. Spacer oligonucleotide typing (spoligotyping), a secondary typing method for MTB strains, has been the gold standard for identifying strains as belonging to the Beijing family, based on absence of spacers 1–34 in the direct repeat (DR) region of the MTB genome [4]. At the same time, another genotyping method that is widely used is the mycobacterial interspersed repetitive unit and variable-number tandem repeats (MIRU-VNTR) method, which can determine the different numbers of mycobacterial interspersed repetitive units with disparate VNTR loci [5]. The combination of the results of the two genotyping methods in a digital format and their discriminatory power, economical cost, and reproducibility facilitates the understanding of MTB epidemiology [4], [5]. In addition, due to the prevalence of the Beijing family in East Asia [6], the former Soviet Union [7], and South Africa [8], research focused on the Beijing genotype has become the hot spot in the tuberculosis field during recent years [9]. The typical subdivision of the Beijing genotype was based upon the analysis of the NTF locus [10] and large sequence polymorphisms (LSPs) [11]. Some Beijing strains were defined as “modern” sublineages (possessing one or two *IS6110* insertions on the right side of the NTF region), the others were “ancient” sublineages (possessing an intact NTF region) [10], [12]. With the exception of the spoligotyping method for Beijing genotype identification, one LSP (RD105) serves as a useful marker for distinguishing the Beijing family, because this LSP was seen in all Beijing strains and additional LSPs (RD181, RD142, and RD 150) could help divide this family further into four monophyletic subgroups [11].

Meanwhile, since the Beijing family was described for the first time in 1995 in Beijing [6], many reports has focused on the prevalence of Beijing family strains in various regions in China, such as Tianjin, Tibet, Jilin, Heilongjiang, and Shanghai [13], [14], [15], [16]. These reports demonstrated that the Beijing family is the most predominant genotype in these provinces; in addition, some other reports have also described the multi-drug resistance (MDR) and high pathogenicity of the Beijing family [17]. In this study, we analyzed the prevalence of the Beijing and other genotypes in clinical MTB strains in Inner Mongolia using the spoligotyping and MIRU-VNTR methods.

## Materials and Methods

### Ethics statement

The study was approved by the Ethics Committee of the National Institute for Communicable Disease Control and Prevention, Chinese Center for Disease Control and Prevention. All patients involved in the study provided written informed consent.

### Mycobacterial strains

**Figure 1:**
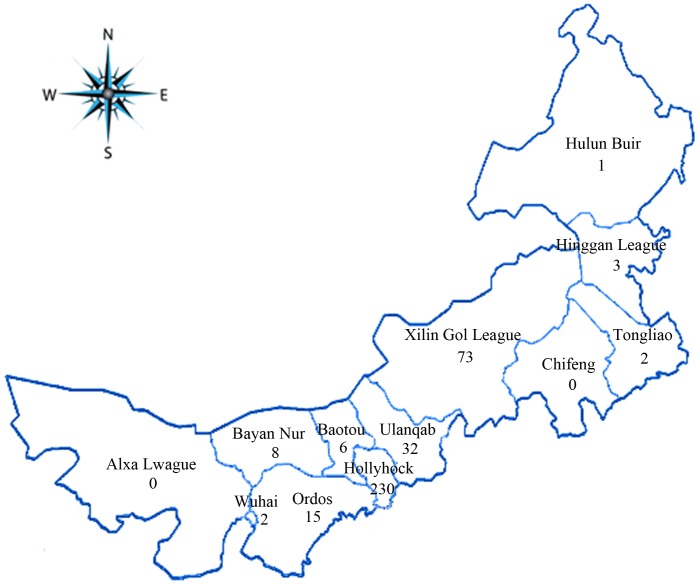
Map of Inner Mongolia showing the distribution of 372 clinical *M. tuberculosis* strains included in this study (the numbers indicate the absolute number of isolates every region).

The study included 372 MTB samples isolated from 372 pulmonary TB patients from various regions of Inner Mongolia whose sputum smears had been diagnosed positive for TB in the Inner Mongolia Chest Hospital in 2011. There were 316 new cases and 56 re-treatment patients. The average age of the patients was 49, and 245 (66.40%, 245/369) patients were male (gender information was available for 369 patients). Among these patients, 230 were from Hollyhock, 6 from Baotou, 2 from Wuhai, 2 from Tongliao, 15 from Ordos, 1 from Hulun Buir, 8 from Bayan Nur, 32 from Ulanqab, 3 from Hinggan League, 73 from Xilin Gol League, and none from Alxa League and Chifeng (Figure 1). MTB H37Rv was used as the reference strain.

### Genomic DNA extraction

Mycobacterial genomic DNA was extracted from mycobacterial colonies grown on Lowenstein-Jensen (L-J) slants. One loop of mycobacterial colonies was resuspended in 400 µl TE buffer (10 mM tris-HCl and 1 mM EDTA) and incubated at 80°C for 30 minutes. Then, the suspension was centrifuged at 12000 rpm for 10 minutes, and the supernatant was stored at −20°C until further use [18].

### Spoligotyping

Spoligotyping of these samples was performed as previously described using the standard protocol described by Kamerbeek et al [4]. First, the direct repeat (DR) region was amplified with the primers DRa and DRb. Then, the PCR products were hybridized to a set of 43 oligonucleotide probes corresponding to each spacer, which were covalently bound to a membrane [4]. Last, the spoligotyes in binary format were compared with those in the SpolDB4 database (http://www.pasteur-guadeloupe.fr:8081/SITVITD).

### MIRU-VNTR

In order to identify a suitable set of MIRU-VNTR loci for genotyping MTB isolates in this area, 22 loci were selected for analysis in the 372 samples. The set of loci included 5 loci of exact tandem repeats (ETRs): ETR-A, -B, -C, -D, and –E; 8 MIRU-VNTRs: MIRU-10, -16, -23, -26, -27, -39, -40, and VNTR3820; 4 Mtub loci: Mtub04, 21, 30, and 39; 5 Queen's University of Belfast (QUB) loci: QUB-11a, -11b, -18, -26, and 4156c. The primers for amplifying each locus have been described in previous studies [5]. Each PCR mixture was prepared in a volume of 12 µl and contained 40 ng of DNA, 6 µl Taq MasterMix (CWBIO, China), 2 µl RNase-Free Water (CWBIO, China), and 1 µl (10 uM) upstream primer and equivalent downstream primer. The amplification cycle was 5 min at 94°C followed by 35 cycles of 45 sec at 94°C, 45 sec at 62°C, and 1 min at 72°C, with a final extension step for 10 min at 72°C. PCR products were analyzed on a 2% agarose gel using a 100-bp DNA ladder (CWBIO, China) as the size marker, the images of the ethidium bromide-stained gels were captured and the copy number of each locus was calculated using Image Lab (Bio-Rad). Then, we used the BioNumerics 5.0 (Applied Maths, St-Martens-Latem, Belgium) software package to complete the phylogenetic and cluster analysis, and determined the discriminatory power of the VNTR loci by means of the Hunter-Gaston discrimination index (HGDI) [19]. The clustering rate was defined using the following formula: clustering rate  =  (*n_c_-c*)/*n*, where *n_c_* is the total number of clustered strains, *c* is the number of clusters, and the *n* is the total number of strains [20].

### LSP and NTF analysis

As mentioned above, NTF and LSP have been used widely as genetic markers of the Beijing family [10], [11]. NTF PCR primers were designed by Sangon Biotech Co. (Shanghai, China). Primer-a was 5′ CCA GAT ATC GGG TGT GTC GAC 3′ and primer-b was 5′ TGC CGT TGT CGA AAT CTA AAC AA 3′. The PCR mixture was prepared as for the MIRU-VNTR analysis, and the amplification cycle was 10 min at 94°C followed by 35 cycles of 1 min at 94°C, 1 min at 66°C, and 1 min at 72°C, with a final step of 10 min at 72°C. Besides NTF, LSP analysis was conducted as previously described [11]. In this paper, we analyzed only RD105 and RD181. After PCR, the products were subjected to electrophoresis on a 2% agarose gel with the BM5000 DNA Marker (BioMed, China) as the size marker. Then, the gels were stained and the images were analyzed according to the method described above [10], [11], [12].

## Results

### Spoligotyping analysis

In this study, we obtained reproducible results from the 372 strains with spoligotyping by referring our data to the SpolDB4.0 database. Among the strains, 294 were clustered into the typical Beijing genotype, 24 were of the Beijing-like genotype, and others (54/372) were of non-Beijing families and subdivided into 37 genotypes. According to the SpolDB4.0 database, 31 strains were not matched, while 341 were successfully clustered by spoligotyping and divided into 8 clusters. Among these identified strains, the most prevalent family was the Beijing family (93.26%, 318/341), followed by the T (5.28%, 18/341), Haarlem family (0.88%, 3/341), LAM (0.29%, 1/341), MANU families (0.29%, 1/341) (Table 1).

**Table 1:** Spoligotypes and RD105 deletion of the *Mycobacterium tuberculosis* isolates (n = 372).

No.	Spoligotype	SIT^a^	Family^b^	RD105 Deletion	N(%)^c^
1	□□□□□□□□□□□□□□□□□□□□□□□□□□□□□□□□□□▪▪▪▪▪▪▪▪▪	1	Beijing	+	294(79.0)
2	□□□□□□□□□□□□□□□□□□□□□□□□□□□□□□□□□□▪▪▪▪▪□▪▪▪	190	Beijing	+	6(1.61)
3	□□□□□□□□□□□□□□□□□□□□□□□□□□□□□□□□□□▪▪▪▪▪▪□▪▪	941	Beijing	+	4(1.07)
4	□□□□□□□□□□□□□□□□□□□□□□□□□□□□□□□□□□▪□▪▪▪▪▪▪▪	621	Beijing	+	2(0.54)
5	□□□□□□□□□□□□□□□□□□□□□□□□□□□□□□□□□□▪▪▪▪▪□□▪▪	541	Beijing	+	1(0.27)
6	□□□□□□□□□□□□□□□□□□□□□□□□□□□□□□□□□□□▪▪▪▪▪▪▪▪	796	Beijing	+	3(0.81)
7	□□□□□□□□□□□□□□□□□□□□□□□□□□□□□□□□□□▪▪▪□▪▪▪▪▪	632	Beijing	+	1(0.27)
8	□□□□□□□□□□□□□□□□□□□□□□□□□□□□□□□□□□▪▪▪▪▪▪▪▪□	NEW	Beijing	+	4(1.07)
9	□□□□□□□□□□□□□□□□□□□□□□□□□□□□□□□□□□▪□▪▪▪▪▪▪□	NEW	Beijing	+	1(0.27)
10	□□□□□□□□□□□□□□□□□□□□□□□□□□□□□□□□□□▪▪▪▪□▪▪▪□	NEW	Beijing	+	1(0.27)
11	□□□□□□□□□□□□□□□□□□□□□□□□□□□□□□□□□□▪▪□□□□□▪□	NEW	Beijing	+	1(0.27)
12	▪▪▪▪▪▪▪▪▪▪▪▪▪▪▪▪▪▪▪▪▪▪▪▪▪▪▪▪▪▪▪▪□□□□▪▪▪▪▪▪▪	53	T1	−	6(1.61)
13	▪□▪▪▪▪▪▪▪▪▪▪▪▪▪▪▪▪▪▪▪▪▪▪▪▪▪▪▪▪▪▪□□□□▪▪▪▪▪▪▪	334	T1	−	4(1.07)
14	▪▪▪▪▪▪▪▪▪▪▪▪▪▪▪▪▪▪▪▪▪▪▪▪▪▪▪▪▪□▪▪□□□□▪▪▪▪▪▪▪	167	T1	−	1(0.27)
15	▪▪▪▪▪▪▪▪▪▪▪▪▪▪▪▪▪▪▪▪▪▪▪▪▪▪▪▪▪▪▪▪□□□□▪▪▪□□□□	51	T1	−	1(0.27)
16	▪▪▪▪□▪▪▪▪▪▪▪▪▪▪▪▪▪▪▪▪▪▪▪▪▪▪▪▪▪▪▪□□□□▪▪▪▪▪▪▪	154	T1	−	1(0.27)
17	▪▪▪▪▪□□□▪▪▪▪▪▪▪▪▪▪▪▪▪▪▪▪▪▪▪▪▪▪▪▪□□□□▪▪▪□▪▪▪	1622	T2	−	2(0.54)
18	▪▪▪▪▪▪▪▪▪▪▪▪▪▪▪▪▪▪▪▪▪▪▪▪▪▪▪▪▪▪▪▪□□□□▪▪▪□▪▪▪	52	T2	−	1(0.27)
19	▪▪▪▪▪▪▪▪▪▪▪▪▪▪▪▪▪▪▪▪▪▪▪▪▪▪▪▪▪▪▪▪□□□□▪▪▪□□▪▪	78	T1-T2	−	1(0.27)
20	▪▪□▪▪▪▪▪▪▪▪▪▪▪▪▪▪▪▪▪▪▪□▪▪▪▪▪▪▪▪▪□□□□▪▪▪▪▪▪▪	1808	T5	−	1(0.27)
21	▪▪▪▪▪▪▪▪▪▪▪▪▪▪▪▪▪▪▪▪▪▪▪▪▪▪▪▪▪▪□▪□□□□▪▪▪□▪▪▪	49	H3	−	2(0.54)
22	▪▪▪▪▪▪▪▪▪▪▪▪▪▪▪▪▪▪▪▪▪▪▪▪▪▪▪▪▪▪□▪□□□□▪▪▪▪▪▪▪	50	H3	−	1(0.27)
23	▪▪▪▪▪▪▪▪▪▪▪▪▪▪▪▪▪▪▪▪▪▪▪▪▪▪▪▪▪▪▪▪□□▪▪▪▪▪▪▪▪▪	54	MANU2	−	1(0.27)
24	□□□□□□□□□□□□□□□□□□□□□□□□▪▪▪▪▪▪▪▪□□□□▪▪▪▪▪▪▪	4	LAM3	−	1(0.27)
25	▪□▪▪▪▪▪▪▪▪▪▪□▪▪▪▪▪▪▪▪▪▪▪▪▪▪▪▪▪▪▪□□□□▪▪▪▪▪▪▪	-	NEW	−	4(1.07)
26	▪▪▪▪▪▪▪▪▪▪▪▪▪▪▪▪▪▪▪▪▪▪▪▪▪▪▪▪▪▪▪▪□□□□▪▪▪□□▪□	-	NEW	−	2(0.54)
27	▪▪▪▪▪▪▪▪▪▪▪▪▪▪▪▪▪▪▪▪▪▪▪▪▪▪▪▪▪□▪▪□□□□▪▪□▪▪▪▪	-	NEW	−	1(0.27)
28	▪□▪▪▪▪▪▪▪▪▪▪▪▪▪▪▪▪▪▪▪▪▪▪▪▪▪▪▪▪▪▪□□□□▪▪□▪▪▪▪	-	NEW	−	1(0.27)
29	▪□▪▪▪▪▪▪▪▪▪□▪▪▪▪▪▪▪▪▪▪▪▪▪▪▪▪▪▪▪▪□□□□▪▪▪▪▪▪▪	-	NEW	−	1(0.27)
30	▪▪▪▪▪▪▪▪▪▪▪□▪▪▪▪▪▪▪▪▪▪▪▪▪▪▪▪▪▪▪▪□□□□▪▪▪□□▪▪	-	NEW	−	1(0.27)
31	▪□▪▪▪▪▪▪▪▪▪▪□▪▪▪▪▪▪▪▪▪▪▪▪▪▪▪▪▪□▪□□□□▪▪▪▪▪▪▪	-	NEW	−	3(0.81)
32	▪▪▪▪▪▪▪▪▪▪▪□▪▪▪▪▪▪▪▪▪▪▪▪▪▪▪▪▪▪▪▪□□□□▪▪▪□□□□	-	NEW	−	1(0.27)
33	▪▪▪▪▪▪▪▪▪▪▪▪▪▪▪▪▪▪▪▪▪▪▪▪▪▪▪▪▪□▪▪□□□□▪□□▪▪▪▪	-	NEW	−	1(0.27)
34	▪▪▪▪▪▪▪▪▪▪▪▪▪□□▪▪▪▪▪▪▪▪▪▪▪▪▪▪▪▪▪□□□□▪▪▪▪▪▪▪	-	NEW	−	1(0.27)
35	□□▪▪□□▪▪□□▪▪□▪□▪▪▪▪□□▪▪▪▪▪□▪▪▪▪□□□▪▪▪▪▪▪▪▪▪	-	NEW	−	2(0.54)
36	□▪▪▪▪▪▪▪▪▪▪▪□▪▪▪▪▪▪▪▪▪▪▪▪▪□▪▪▪▪▪□□□▪▪▪▪▪▪▪▪	-	NEW	−	1(0.27)
37	▪▪▪▪□▪▪▪▪□□□□□□□▪▪▪▪▪▪▪▪▪▪▪▪▪▪▪▪□□□□▪▪▪▪□□□	-	NEW	−	1(0.27)
38	▪▪▪▪▪▪▪▪▪▪▪□▪▪▪▪▪▪▪▪▪▪□□□□□□▪▪▪▪□□□□▪▪▪□▪▪□	-	NEW	−	1(0.27)
39	▪□□□□□□▪▪▪▪□□□▪▪▪▪▪▪▪▪▪▪▪▪▪▪▪▪▪▪□□□□▪▪▪▪▪▪□	-	NEW	−	1(0.27)
40	▪▪▪▪▪▪▪▪▪▪▪□▪▪▪▪□▪▪▪▪▪▪▪▪▪▪▪▪▪▪▪□□□□▪▪▪▪▪▪□	-	NEW	−	1(0.27)
41	▪▪▪▪▪▪▪▪▪▪▪□□□□□□□□□□□□□□□□□□□▪▪□□□□▪□▪▪▪▪▪	-	NEW	−	1(0.27)
42	▪□▪▪▪▪▪▪□□▪□▪▪▪▪□▪▪▪▪▪▪▪▪▪▪▪▪▪▪▪□□□□▪▪▪▪▪▪□	-	NEW	−	1(0.27)
43	▪▪▪▪▪▪▪▪▪▪▪□▪▪▪▪□▪▪▪▪▪▪▪□□□□□□□▪□□□□▪▪▪□▪▪▪	-	NEW	−	1(0.27)
44	□▪▪▪▪▪▪▪▪▪▪▪▪▪▪▪▪▪▪▪▪▪▪▪□□□□□□□□□□□□□□□□□□□	-	NEW	−	1(0.27)
45	□□▪▪▪▪▪▪▪▪▪▪▪▪▪▪▪▪▪▪▪▪▪▪▪▪▪▪▪▪▪▪□□□▪▪▪▪□▪▪▪	-	NEW	−	1(0.27)
46	▪▪▪▪▪▪▪▪▪▪▪▪▪▪□□□▪□□□□□□▪▪▪▪▪▪▪▪□□□□▪▪▪□▪▪▪	-	NEW	−	1(0.27)
47	□□▪▪□□▪▪▪▪▪▪□▪□▪▪▪▪□▪▪▪▪□□□□□□□□□□▪▪▪▪▪▪▪▪▪	-	NEW	−	1(0.27)
48	□▪▪▪▪▪▪▪▪▪▪▪▪▪▪▪▪▪▪▪▪▪▪▪□□□□□□□□□□▪▪▪▪□□□▪□	-	NEW	−	1(0.27)

a SIT number from the Spoldb4.0 database. SIT, spoligotype international type.

b Spoligotype families as assigned in Spoldb4.0.

c The number of the isolates with a common SIT.

### 22-Loci MIRU-VNTR analysis

**Figure 2:**
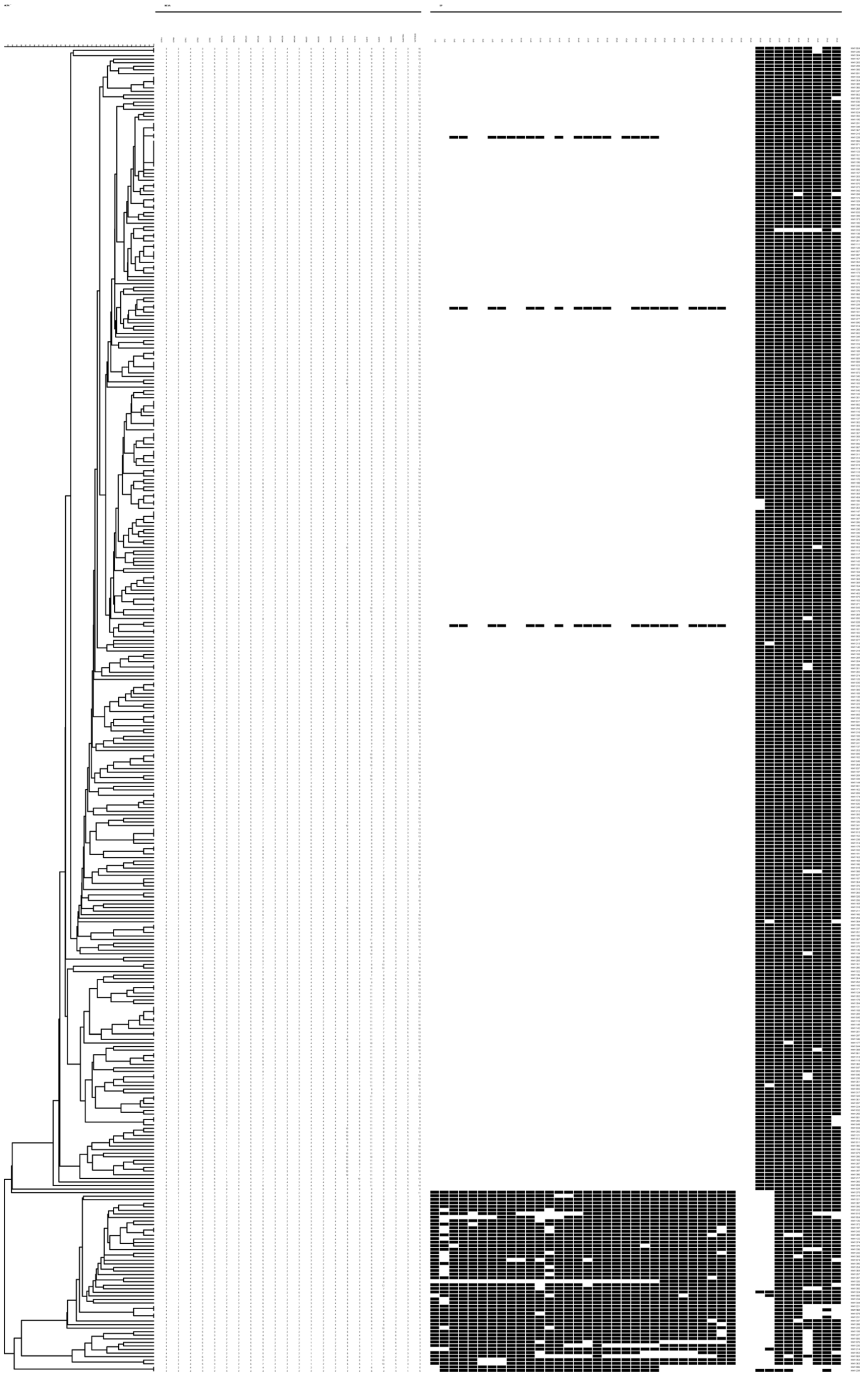
Genotyping of 372 MTB isolates with 22-loci MIRU-VNTR and spoligotyping. The clustering was based on the analysis performed using BioNumerics 5.0 to estimate the results of these two genotyping methods.

The 372 isolates were genotyped using 22 MIRU-VNTR loci (ETRA, ETRB, ETRC, ETRD, ETRE, MIRU10, MIRU16, MIRU23, MIRU26, MIRU27, MIRU39, MIRU40, Mtub21, Mtub30, and Mtub39), and 308 different VNTR genotypes were detected. Two hundred and sixty-one types represented single isolates and 47 genotypes had 2 or more strains (Figure 2). Further analysis of phylogenetic clustering and genotypic characteristics using the BioNumerics 5.0 indicated that the clustering rate was 17.20%. Obviously, the Beijing family was the largest group among these strains.

Next, the 22-loci MIRU-VNTR genotyping showed different discriminatory powers of the loci. The Hunter-Gaston discriminatory index (HGDI) scores varied significantly from 0.838 for VNTR3820 to 0.068 for MIRU23 (Table 2). As previously reported [21], the MIRU loci were further classified into highly (>0.6), moderately (0.3 to 0.6), and poorly (<0.3) discriminatory based on the HGDI scores. In this study, the discriminatory power of 6 loci (VNTR3820, Qub11b, Qub18, Qub11a, Mtub21 and Qub26) was higher than 0.6, supporting their designation as highly discriminatory loci, 8 loci (MIRU26, Mtub04, ETRA, ETRE, MIRU10, Mtub30, MIRU39, and Qub4156c) showed moderate discriminatory power, and the others were found to be less polymorphic. The specific discriminatory powers of these loci are shown in Table 2.

**Table 2:** HGDI scores of the 22 MIRU-VNTR loci for all the samples and the Beijing family strains.

	Alias	HGDI score	HGDI score
		All strains	Beijing family
1	VNTR3820	0.838	0.792
2	Qub11b	0.756	0.702
3	Qub18	0.706	0.629
4	Qub11a	0.663	0.627
5	Mtub21	0.655	0.574
6	Qub26	0.624	0.572
7	MIRU26	0.548	0.437
8	Mtub04	0.461	0.319
9	ETRA	0.425	0.312
10	ETRE	0.402	0.238
11	MIRU10	0.388	0.244
12	Mtub30	0.334	0.149
13	MIRU39	0.327	0.132
14	Qub4156c	0.316	0.331
15	MIRU40	0.299	0.221
16	Mtub39	0.285	0.188
17	MIRU16	0.204	0.126
18	ETRB	0.194	0.037
19	ETRD	0.134	0.086
20	ETRC	0.127	0.136
21	MIRU27	0.083	0.025
22	MIRU23	0.068	0.037
23	Cumulative HGDI	0.9902	0.9982

MIRU, mycobacterial interspersed repetitive unit; VNTR, variable-number tandem repeats; Qub, Queen's University of Belfast; ETR, exact tandem repeats; Mtub, Mycobacterial tuberculosis; HGDI, Hunter-Gaston Discriminatory Index.

### Comparison between Spoligotyping and 22-Loci MIRU-VNTR

As showed in Figure 2, there was a difference between the MIRU-VNTR and spoligotyping analyses of the Beijing family, in that 3 non-Beijing family strains were clustered in the Beijing family. Because of this, based on the fact that Beijing family strains accounted for 85.48% (318/372) of cases in this study, we compared the results of the two methods in clustering of Beijing family strains. As displayed in Table 1, 11 subtypes of the Beijing family were detected in these 318 Beijing family strains, and the HGDI score was 14.49%. Meanwhile, by the 22-loci MIRU-VNTR analysis, there were 260 genotypes and 41 clusters in the 318 strains, with an HGDI score of 99.82%. Apparently, the discriminatory power of the 22-loci MIRU-VNTR method was higher than that of spoligotyping in analyzing the Beijing family.

### NTF and LSP analysis

The NTF analysis of the Beijing family strains showed that 244 (244/318) samples had one or two *IS6110* insertions in the NTF region, and were designated as “modern” sublineage. The remaining strains (74/318) had an intact NTF region, and were designated as “ancient” sublineage.

In the LSP analysis of 372 isolates, there were 318 strains lacking RD105, and the other 54 strains had a RD105 fragment. The results of the RD105 analysis were very similar with that of the spoligotyping for identification of the Beijing family strains. The correlation between the LSP and spoligotyping data is presented in Table 1. Moreover, analysis of RD181 showed that the proportion of RD181[+] and RD181[-] samples were 8.18% (26/318) and 91.82% (292/318), respectively. Also, the Beijing family strains that had an intact RD181 were all included in the “ancient” sublineage.

### Relationship between the Beijing family and the nationalities

We estimate the relationship between the Beijing genotype and two nationalities, Han and Mongol nationalities, which were the two main nationalities of the people in Inner Mongolia (Table 3). According to the chi-square test, we find that there is no correlation between the Beijing family distribution and these two main nationalities (P>0.05).

**Table 3:** Statistical analysis of the correlation between the Beijing genotype and the nationalities.

Nationality	Beijing genotype (No. of samples)	Non-Beijing genotype (No. of samples)
Han	248	34
Mongol	67	17

χ2 = 3.612, P = 0.057; P>0.05.

## Discussion

The results of this study show that the Beijing family is the most prevalent lineage of MTB strains in Inner Mongolia. Although there are no previous reports of MTB genotyping in Inner Mongolia, these results are consistent with those of other papers which demonstrated that the Beijing genotype is the most predominant genotype in China, in regions such as Tianjin (91.7%), Tibet (90.63), Jilin (89.9%), Heilongjiang (89.5%), and Shanghai (89%) [13], [14], [15], [16]. Moreover, the proportion of the Beijing family in Inner Mongolia conforms to that of former reports, in that the highest prevalence of the Beijing family was found in northern China, followed by central and southern China [15].

A comparison of the results of the spoligotyping and the RD105 analysis indicated that the identification of the Beijing family was consistent, and that the isolates containing at least 3 spacers among the 35 to 43 direct repeats lacked RD105. So, these two methods are useful for identifying the Beijing family. Furthermore, to analyze the evolution of the Beijing family we analyzed the *IS6110* insertions in the NTF region. The results show that 76.73% of the Beijing family strains belong to the “modern” group, which is nearly the same as previously reported (76.6%) [22]. Although there are no other published reports addressing the evolution of the Beijing family by NTF analysis in China, we can assume that the proportion of the “modern” group is larger than that of the “ancient” sublineages, and more research will be needed to confirm this. Concomitantly, all the RD181[+] strains were included in the “ancient” sublineage, and the other “ancient” strains were RD181[-]. These results show that not all “ancient” strains have intact RD181, from which we can infer that the RD181 deletion happened before the insertion of *IS6110* in the NTF region [23], [24].

Spoligotyping has been considered as the gold standard for Beijing family identification because it is simple and efficient. However, the discriminatory power of spoligotyping is low and it cannot be utilized for further analysis of the Beijing family strains. As shown in this study, there were 48 genotypes, 15 clusters, and 33 unique spoligotypes. Owing to the shortage of spoligotypes, we utilized another molecular typing method based on MIRU-VNTR which has been used in many epidemiological studies [14], [16], [25]. In the present study, 308 genotypes, 47 clusters, 261 unique genotypes, and a HGDI score of 99.02% was obtained by MIRU-VNTR analysis. In addition, the cumulative HGDI score (14.49%) of the 22-loci MIRU-VNTR was lower than that of spoligotyping (99.82%) in the genotyping of the Beijing family strains. The obvious difference between the results of spoligotyping and MIRU-VNTR indicates that both methods have some advantages, but their combination yields additional information and can facilitate molecular epidemiologic analysis of MTB. Therefore, the use of both these methods would be the most suitable for genotyping analysis of MTB strains.

In different areas, the disparate VNTR typing sets showed various efficiencies in MTB genotyping. The sets of common loci are comprised of 12 loci, 15 loci, 24 loci, and others [5], [25], [26], and the discriminatory power of MIRU-VNTR is determined by the number of loci. As indicated in some of the latest studies, Japan (JATA-VNTR), Nigeria, and Northwestern Russia utilized JATA-VNTR, 24 VNTRs, and 15 VNTRs (12 VNTRs + 3 hypervariable loci), respectively, to differentiate their isolates and the Beijing strains [27], [28], [29]. The suitable sets of loci depend on the population structure of MTB in the investigated area. In present study, the 22 loci scheme was chosen based on the available data for other provinces in China [13], [14], [15], [16], [21]. In addition to spoligotyping, the 22-loci VNTR also can efficaciously analyze the Beijing family (Table 2).

Based on the statistical data [30], the proportions of the Han and Mongol nationalities were about 78% and 18%, respectively, in 2007 in Inner Mongolia, and the proportion of the Mongol nationality has been constantly rising in recent years. In the present study, the proportions of the Han and Mongol nationalities were 75.81% and 22.58%, respectively. The other nationalities were not considered in this analysis because of their insignificant contributions to the total population. Therefore, the analysis of the Beijing family and the two major nationalities is likely effective in delineating the correlation between nationality and MTB strain genotypes. In addition, the samples from different regions of Inner Mongolia were not uniformly distributed, so we cannot discuss the association between the geographic characteristics and the TB epidemic situation.

## Conclusion

In conclusion, the data in this study indicate that the Beijing family is the predominant genotype in Inner Mongolia, and that the Beijing family is not correlated with the nationalities in this area. Further studies addressing the association between different regions and the epidemiology of MTB are needed to better understand the molecular epidemiology of MTB.

## Supporting Information

Table S1 **22-loci MIRU-VNTR profile, spoligotyping profile, NTF analysis and LSP (RD105, RD181) results of 372 isolates.** The table provides the typing data obtained using the 22-loci MIRY-VNTR analysis and spoligotyping, as well as the results of the NTF and LSP analysis of the 372 isolates.
(XLSX)
